# Chemotherapy and Other Systemic Drugs Used to Treat Gynecologic Carcinosarcomas (GCSs): A Retrospective Analysis from Hospital Clínico San Carlos (HCSC), An Academic Referral Centre for Rare Gynecological Malignancies in Madrid, Spain

**DOI:** 10.3390/cancers17203359

**Published:** 2025-10-17

**Authors:** Gloria Marquina, Beatriz Gonzalez-Diez, Pluvio J. Coronado, Javier Garcia Santos, Mar Ramirez, Monica Bellon, Rafael Sanchez del Hoyo, Alejandro Pascual, Cristina Diaz del Arco, Noelia Sanmamed Salgado, Elena Cerezo, Ramiro Mendez, Miguel Muñoz, Jose Manuel Espejo, Angel Nava, Susana Martin Garre, Cristina Rodriguez, Aida Ortega, Antonio Casado

**Affiliations:** 1 School of Medicine, Universidad Complutense de Madrid, IdISSC, EURACAN Referral Center, 28040 Madrid, Spain; 2 Department of Medical Oncology, Hospital Clinico San Carlos, 28040 Madrid, Spain; 3 Department of Obstetrics and Gynecology, Hospital Clinico San Carlos, School of Medicine, Universidad Complutense de Madrid, IdISSC, EURACAN Referral Center, 28040 Madrid, Spain; 4 Research Methodological Support Unit and Preventive Department, Hospital Clinico San Carlos, IdISSC, 28040 Madrid, Spain; 5 Department of Pathology, Hospital Clinico San Carlos, School of Medicine, Universidad Complutense de Madrid, IdISSC, EURACAN Referral Center, 28040 Madrid, Spain; 6 Department of Radiotherapy, Hospital Clinico San Carlos, School of Medicine, Universidad Complutense de Madrid, IdISSC, EURACAN Referral Center, 28040 Madrid, Spain; 7 Department of Radiology, Hospital Clinico San Carlos, School of Medicine, Universidad Complutense de Madrid, IdISSC, EURACAN Referral Center, 28040 Madrid, Spain; 8 Department of Nuclear Medicine, Hospital Clinico San Carlos, School of Medicine, Universidad Complutense de Madrid, IdISSC, EURACAN Referral Center, 28040 Madrid, Spain

**Keywords:** gynecologic carcinosarcomas, rare gynecological tumors, systemic therapy, chemotherapy, immunotherapy, genital neoplasms, female, uterine cancer

## Abstract

Background/Objectives: Gynecologic carcinosarcomas (GCSs) are rare cancers with poor prognoses. In this study, we aimed to analyze the chemotherapy regimens used to treat GCSs at our institution, as well as the patients’ outcomes. Methods: We conducted a retrospective analysis of 62 GCS patients treated at Hospital Clinico san Carlos (HCSC) from 1995 to 2024. Results: Data from 62 GCSs were analyzed. Fifty-two patients (84%) underwent surgery at diagnosis, followed by radiotherapy in 20 (38.5%) endometrial carcinosarcoma (ECS) patients and perioperative chemotherapy in 45 GCS patients (62.6%). After a median follow-up (mFollow-up) of 20 months (0–242 months), 23 GCS patients relapsed and 13 progressed after the first treatment approach, 3 of them almost immediately after surgery. The median time to disease relapse was 6 months (0–225 months) and the median time to disease progression was 0 months (0–25 months). Sixteen of the relapsing patients and fifteen of the patients who were diagnosed at stage IV received systemic treatment with carboplatin and paclitaxel, the most common forms of chemotherapy (used as first-line treatments in eight of the relapsing patients and in seven patients who were in stage IV at diagnosis). GCS patients received up to five lines. The median progression-free survival (mPFS) was 5 months for the first line, and decreased in the following lines. The median overall survival (mOS) was 24 months (13.5–34.5 months) and 11 months (1–226 months) after GCS relapse/progression. Conclusions: GCS is a rare and aggressive disease with a high recurrence rate after surgery and short progression-free survival (PFS) at advanced stages, even when a combination of oncological therapies are used. (Nevertheless, some patients achieved long disease-free survival). To the best of our knowledge, this is the first publication to provide this valuable data on this rare and aggressive disease, for which phase III and real-world data is scarce.

## Simple Summary

This retrospective study evaluated 62 patients with gynecologic carcinosarcomas (GCSs) treated at Hospital Clínico San Carlos (Madrid, Spain) between 1995 and 2024. Most patients underwent surgery followed by platinum-based chemotherapy (mainly carboplatin and paclitaxel) and, in some cases, radiotherapy. Despite this multimodal approach, outcomes remained poor, with a high recurrence rate (58%), a median progression-free survival of 5 months, and a median overall survival of 24 months. This study presents the first real-world evidence on the disease control rate, mPFS, and mOS of second and subsequent treatment lines in GCS patients, including information about the treatments administered in each line. These findings confirm the aggressive nature and limited treatment options for GCS, highlighting the urgent need for further research and novel therapies.

## 1. Introduction

Gynecologic carcinosarcomas (GCSs) are rare cancers that were formerly classified and treated as sarcomas. Since the early 2000s, GCSs have been known to be metaplastic carcinomas with molecular features that overlap adenocarcinomas [1], characterized by the presence of both carcinomatous and sarcomatous components. They account for less than 5% of uterine and ovarian malignancies [2,3], with a poor prognosis, with a 5-year overall survival (OS) of less than 30% and median OS (mOS) of less than two years [4]. 

GCSs mainly arise in the uterus and the ovaries. Ovarian CS (OCS) is currently considered by definition and treated as a high-grade ovarian carcinoma [5], and endometrial CS (ECS) is treated as an aggressive histological type of endometrial carcinoma [6]. In early and locally advanced stages, surgery is the backbone treatment, followed by adjuvant chemotherapy (AdjChT) in OCS patients or chemoradiotherapy in patients with ECS. In metastatic disease, the standard treatment is chemotherapy (ChT), but there is a lower response rate and poorer prognosis compared to those observed for other high-grade carcinomas [7]. A few ECS patients have recently been included in a phase III study in which immunotherapy was added to the standard platinum-based ChT, with promising results [8]. 

GCS are not usually included or well represented in clinical trials; therefore, real-world information about the use of systemic treatment in this rare disease is key. Thus, we describe our experience treating GCSs at Hospital Clínico San Carlos (HCSC), Madrid (Spain), as part of the European Reference Network on Rare Adult Cancers (EURACAN) referral center for rare gynecological malignancies, and discuss patients’ outcomes in terms of survival. 

## 2. Materials and Methods

Data were collected retrospectively from patients’ physical and electronic medical records from HCSC. The data of consecutive patients with pathologically confirmed GCS were included. Clinical characteristics included age, menopausal status at diagnosis, International Federation of Gynecology and Obstetrics (FIGO) stage 2009 and 2023 for ECS [9,10] and 2014 for OCS [11], Eastern Cooperative Oncology Group (ECOG) performance status, primary tumor (uterine or ovarian), CA125 at diagnosis, ascites at diagnosis, primary treatments (surgery ± adjuvant radiotherapy (AdjRT) ± AdjChT), date of relapse (if any), sites of relapse (pelvic/ lymph nodes/ peritoneal, lung, brain, bone, and other visceral sites), number of lines administered, and the type of ChT administered at every line. ECS stages I and II were analyzed together as FIGO 2023 upstaged stage I ECS to stage II. The year of diagnosis was compiled as <2003 and ≥2003 when systemic treatment shifted from sarcoma to endometrial cancer regimens.

Qualitative variables are presented with their frequency distribution. Quantitative variables are summarized with their median and their range. Survival was studied using descriptive Kaplan–Meier graphs. The log-rank test was used to compare survival curves. A significance value of 5% was accepted for all tests. Data processing and analysis were performed using IBM SPSS Statistics v.26 statistical software and R v.4.5.1 and packages: dplyr (function “%>%” and “filter”); haven (function “read_sav”); survival (function “survfit” and “Surv”); and survminer (function “ggdurvplot”).

Overall survival (OS) was defined as the total length of time from diagnosis until the patient’s death, regardless of the cause of death. Progression-free survival (PFS) was defined as the length of time from the end of a treatment until the first evidence of disease progression or death from any cause. Time to disease relapse was defined as the total length of time (in months) from the end of a treatment until the first evidence of disease relapse. Time to disease progression was defined as the total length of time (in months) from the end of a treatment until the first evidence of disease progression.

## 3. Results

### 3.1. Patient Characteristics

Sixty-two GCS patients were treated at HCSC from 1 January 1996 to 31 December 2024, 49 ECS (79%) and 13 OCS (21%). The mean age at diagnosis was 66 (30–90), with most patients being postmenopausal (98%). Abnormal vaginal bleeding was the most commonly presenting symptom, and was reported in 50% of the patients. Information about CA125 was available for 45 patients, and it was found to be abnormal in 28 of them. The median serum CA125 at diagnosis was 48 IU/mL (5–2130 UI/mL); the upper limit in our laboratory is 35 IU/mL The main patient characteristics are summarized in Table 1.

### 3.2. Multidisciplinary Management as a First Treatment Approach

The information about the multidisciplinary management is summarized in Table 2. Most patients—52 (84%)—underwent surgery at diagnosis with the following FIGO stages: I, 24 patients; II, 6 patients; III, 15 patients; and IV, 7 patients.

AdjRT was administered to 20 ECS patients (32.25%). All these patients received brachytherapy; in addition, 16 patients underwent pelvic radiotherapy (RT) (25.8%) and 1 patient underwent paraaortic RT (2%). None of the OCS patients received AdjRT.

Forty-five patients (62.6%) received perioperative ChT as part of the initial multidisciplinary management. Platinum-based ChT was the AdjChT administered in most of the patients, with carboplatin and paclitaxel being the most frequent combination (administered to 23 patients); other AdjChT regimens involved administration of cisplatin and ifosfamide (6 patients) or carboplatin and epirubicin (2 patients). 

After the first treatment approach, 35 patients (56.4%) achieved a complete response, 1 (1.6%) had stable disease, 23 (37%) exhibited objective disease progression and 3 (4.8%) were not evaluated due to death caused by the GCS. More specifically, at the time of data analysis, the statuses of patients treated with neoadjuvant chemotherapy (NACT) or AdjChT (45 patients) were as follows: 16 (35.5%) were alive without disease relapse, 14 (31.1%) experienced a disease relapse, and 15 (33.3%) died. 

### 3.3. Disease Relapse

At a median follow-up (mFollow-up) of 20 months (0–242 months), 36 patients (58%) relapsed or progressed (23 relapses and 13 disease progressions) after the first multidisciplinary management or first-line treatment, respectively. The median time to disease relapse was 6 months (0–225 months) and was slightly different between ECS and OCS: 6 months (0–225 months) vs. 5 months (0–154 months), respectively. The time to disease relapse also differed between FIGO stages: stage I and II: 52 months (0–225 months); stage III: 6 months (0–161 months). As for stage IV GCS, the median time to disease progression was 0 months (0–25 months).

The number of relapses differed between FIGO stages at diagnosis. Table 3 describes the frequency of relapses according to the FIGO 2009 stage at diagnosis and the status of the patients at the time of data analysis.

At the time of relapse/progression, 19 patients (52.7%) were treated with ChT only, 1 patient underwent surgery to treat lung relapse, and 1 patient (2.7%) received palliative RT in response to bone progression. Other treatment approaches at the time of disease relapse/progression were as follows: two patients (5.5%) received ChT and RT, two patients (5.5%) underwent surgery followed by ChT, and two patients (5.5%) received the best supportive care.

The sites of disease relapse were as follows: pelvis (17 patients (47.2%)), peritoneal (13 patients (36.1%)), lymph nodes (6 patients (16.6%)), other visceral locations (5 patients (13.8%)), lung (4 patients (11.1%)), bone (2 patients (5.5%)), and brain (1 patient (2.7)).

### 3.4. Systemic Treatment in the First and Following Lines

Twenty-six patients (41.9%) were treated in the advanced setting, receiving first-line treatment with a median progression-free survival (mPFS) of 5 months (95% confidence interval 95% (95% CI) 4.2–5.8) and an mOS of 12 months (95% CI 6–18). Twenty patients (76.9%) received a second-line treatment and had mPFS of 2 months (95% CI 0–5). Ten patients (38.4%) received third-line treatment with an mPFS of 2 months (95% CI 1–3). Four lines of treatment were administered in six patients (23%), with an mPFS of 2 months (95% CI 0–4.4). The outcome of each of the treatment lines is shown in Table 4. The ChT regimens used in treatment along with the mPFS achieved are shown in Table 5. 

### 3.5. Overall Survival

**Figure 1:**
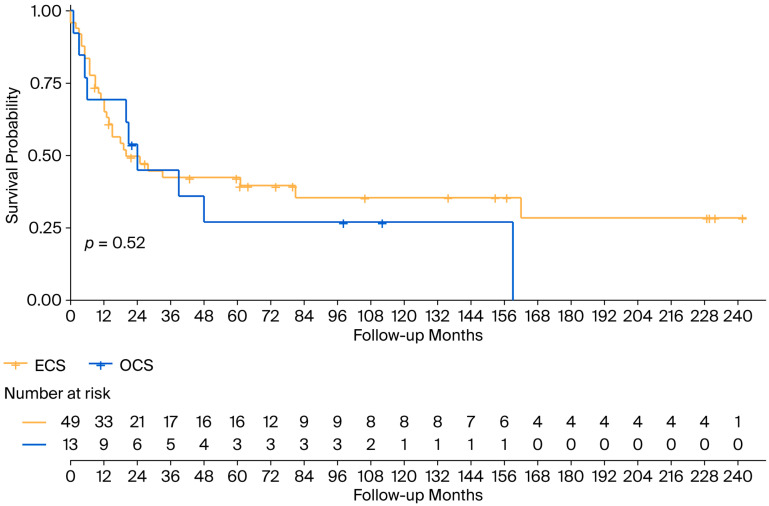
Overall survival in the ECS and OCS cohorts. ECS: endometrial carcinosarcoma; OCS: ovarian carcinosarcoma.

**Figure 2:**
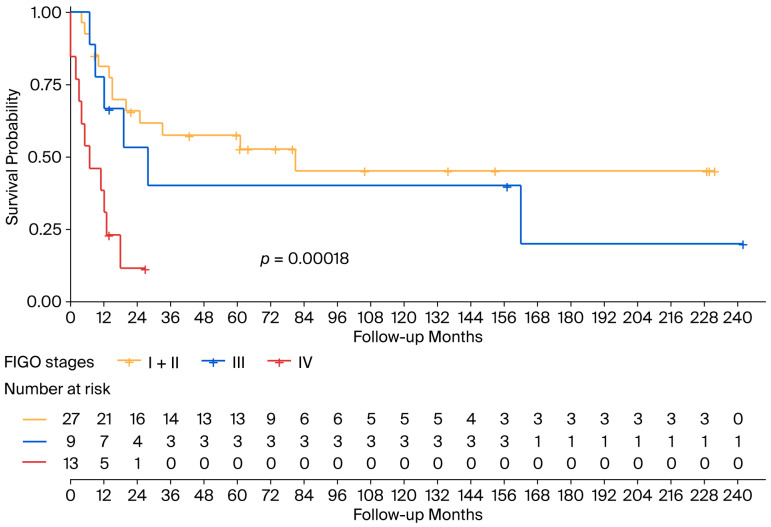
Overall survival according to FIGO stage in the ECS population. ECS: endometrial carcinosarcoma; FIGO: International Federation of Gynecology and Obstetrics.

With an mFollow-up of 77 months from diagnosis (9–242 months), the mOS was 24 months (95% CI 13.5–34.5): 20 months (95% CI 4–36) in the ECS cohort and 24 months (95% CI 17.4–30.7) in the OCS patients (Figure 1). Regarding survival according to FIGO staging, the mOS among patients with ECS was 81 months in patients in FIGO stage I-II, 28 months (95% CI 7.6–48.4) for patients in stage III, and 7 months (95% CI 0–15.2) for patients in stage IV (Figure 2).

The median survival after GCS relapse/progression was 11 months (1–226 months). It differed between ECS and OCS: 7 months (0–226 months) vs. 11 months (0–36 months). The 3-year overall survival rate was 16.7% in the ECS cohort and 11.1% in the OCS cohort. 

At the time of data analysis in December 2024, 20 patients (32.3%) were alive without disease recurrence, 2 patients (3.2%) were alive with disease, and 40 (64.5%) patients were dead.

## 4. Discussion

In this paper, we show real-world evidence of the initial multidisciplinary management along with a thorough description of the systemic therapies used at relapse and the outcomes of the regimens at each line of a cohort of 62 GCS patients treated at a EURACAN referral center for rare gynecological malignancies. The cohort was enriched with patients from 2003 onwards, with only 10 patients treated before the early 2000s; thus, the treatment of most of the patients is of the current standard.

Fifty-two of the sixty-two GCS patients in our cohort underwent surgery, with twenty of the ECS receiving adjRT and forty-five patients (62.6%) receiving perioperative ChT. This multimodal approach (surgery + adjRT + ChT) is the current standard in locally advanced ECS. Platinum-based ChT was the perioperative ChT regimen administered in most of the patients, with carboplatin and paclitaxel [13,14] being the most frequent combination (23 patients), accounting for a better toxicity profile and non-inferior outcomes than other regimens administered, which were cisplatin and ifosfamide (6 patients) [15] or carboplatin and epirubicin (2 patients). Several studies focusing on newly diagnosed ECS patients also used carboplatin and paclitaxel [16,17]. However, only Hoskins et al.’s publication contains information on the disease relapse rate (33%) [16], which is similar to our study (31%). Two studies mention the different ChT regimens given to GCS patients, but they do not provide any information about the outcomes of the lines or the regimens used [18,19].

In our study, 26 patients were treated [13,14] in the first-line setting. Carboplatin and paclitaxel was the most common ChT regimen used (administered to 12 patients) followed by platinum-based ChT in 9 patients. Years ago, a small number of sarcoma patients were treated with first-line ChT regimens such as doxorubicin and ifosfamide, gemcitabine and adriamycin, gemcitabine and docetaxel, gemcitabine and dacarbazine, or cisplatin and cyclophosphamide. None of the patients were treated with the triplet cisplatin, doxorubicin and ifosfamide, which was tested years ago with a high toxicity profile [20]. Carboplatin and paclitaxel is the current first-line standard combination in GCS due to its better toxicity profile and non-inferiority activity compared to the prior first-line standard, paclitaxel and ifosfamide, as shown in GOG-232B [21] and GOG-261 [22]. Our cohort showed a shorter mPFS and mOS compared to GOG-261 (5 vs. 16 months and 20 vs. 37 months, respectively). A possible explanation for the difference in mPFS is that our cohort included recurrent patients with prior exposure to carboplatin and paclitaxel; as for the difference in mOS, only half of the patients in our cohort (46%) received carboplatin and paclitaxel as a first-line treatment. Notably, our cohort includes patients treated with this combination in subsequent lines—three in the third-line treatment and one in the fifth-line treatment, with an mPFS of 1 month and 5 months, respectively—thus showing activity in further lines. Carboplatin and paclitaxel have shown improved activity in this rare disease in response to the addition of a novel promising drug: dostarlimab [8]. Two patients in our cohort received this triplet treatment, with an mPFS of 17 months. Specific data of only the 25 ECS patients included in the RUBY trial have not been published, but the results for the entire endometrial carcinoma population included showed a 24-month PFS of 56.8% [23]. A patient in our study with recurrent OCS received maintenance niraparib, a poly (ADP-ribose) polymerase (PARP) inhibitor, in line with other high-grade recurrent ovarian cancers.

Reports in the literature on second- and subsequent-line regimens in GCS are scarce. Cisplatin and ifosfamide have been studied in phase II and phase III studies in GCS patients, showing an mPFS between 2 and 4 months in phase II [24] and 6 months in phase III [25]. In our cohort, two patients received this regimen with an mPFS of 5 months, in line with the clinical trial data. Gemcitabine and docetaxel have been tested in a phase II study in second-line recurrent GCS, showing mPFS of 1.8 months [26]. Our data for two patients receiving this regimen are 1 month—in line with phase II—and 226 months—longer than in the phase II study. Another combination used in our patients is carboplatin and pegylated liposomal doxorubicin, which was tested in a phase II study mainly in newly diagnosed GCS patients. Only 1 of the 20 GCS patients had recurrent disease, showing an mPFS of 9.4 months, whereas in our cohort, the 2 patients who underwent second-line treatment and 1 patient who received fourth-line treatment had an mPFS of 5.5 and 4 months, respectively.

Table 6 summarizes the publications on real-world evidence of systemic treatment in GCS to date. There are notably significant differences between any of these published cohorts and ours: fewer patients were included in previously published cohorts [16,19,27,28]; most of these publications do not specify the ChT regimens used not only in the first line but also in subsequent lines; only one publication appropriately describes the outcomes of the first-line therapy [16]; and all but two [19,29] of these studies had a shorter median follow-up than our 29-year cohort.

As far as we are concerned, this is one of the largest GCS studies published in the literature, and it is unique as it includes a long-term follow-up of the patients and specifies the chemotherapy regimens used in each line and the outcomes in terms of survival and response rate, along with the results depending on the FIGO stage and the incorporation of new drugs such as dostarlimab. This publication has limitations, mainly due to the retrospective nature of the data collection and analysis and the treatment variability within patients.

## 5. Conclusions

To the best of our knowledge, this study presents the first real-world evidence on the disease control rate, mPFS, and mOS of second and subsequent treatment lines in GCS patients, including information about the treatments administered in each line. GCS is a disease for which phase III and real-word data are scarce. In the advanced stages, these patients have a dismal prognosis. Further research is needed to improve the outcomes of GCS patients. 

## Figures and Tables

**Table 1:** Main patient characteristics.

Characteristics	Number of Patients (%)
**Menopausal** **s** **tatus**	
Premenopausal Postmenopausal	1 (1.6) 61 (98.4)
**ECOG**	
0 1 ≥2	36 (58.1) 17 (27.4) 9 (14.5)
**Primary disease**	
Uterus Ovary	49 (79.1) 13 (20.9)
**Ascites at diagnosis**	
Present Absent	9 (14.5) 53 (85.5)
**FIGO 2009 stage at diagnosis (ECS)**	
I II II IV	22 (44.9) 5 (10.2) 9 (18.4) 13 (26.5)
**FIGO 2023 stage at diagnosis (ECS)**	
II 1II IV	27 (55.1) 9 (18.4) 13 (26.5)
**FIGO 2014 stage at diagnosis (OCS)**	
I	2 (15.4)
II	3 (23.1)
III	6 (46.1)
IV	2 (15.4)
**Year of diagnosis**	
<2003 ≥2003	10 (16.1) 52 (83.9)

ECOG: Eastern Cooperative Oncology Group; FIGO: International Federation of Gynecology and Obstetrics.

**Table 2:** Multidisciplinary management.

Treatment	Number of Patients (%)
**Primary surgery**	
No surgery Surgery R0 R1 R2	10 (16.1) 52 (83.9) 43 (82.7) 2 (3.8) 7 (13.5)
**ChT**	
No AdjChT NACT First line	17 (27.4) 31 (50) 3 (4.8) 11 (17.8)
**ChT agents**	
Carboplatin + paclitaxel Other	30 (66.7) 15 (33.3)
**AdjRT (ECS only)**	
Yes No	20 (40.8) 22 (44.9)

AdjChT: Adjuvant Chemotherapy; AdjRT: Adjuvant Radiotherapy; ChT: Chemotherapy; NACT: Neoadjuvant Chemotherapy.

**Table 3:** GCS disease relapse, patient status, and mFollow-up according to FIGO stage.

Stage	N	Relapsed (%)	Alive Without Relapse	Alive with Disease	Death	mFollow-Up
I	24	10 (41.7%)	13	1	10	61 (5–232)
II	8	6 (75%)	2	0	6	12.5 (3–80)
III	15	8 (53.3%)	4	0	11	22 (1–242)
IV	15	13 (86.6%)	1	1	13	7 (0–27)

mFollow-Up: Median Follow-Up; N: Number of Patients.

**Table 4:** Outcome of each line of treatment evaluated by RECIST v1.1 [12] and by mPFS.

Line of Treatment	N	CR	PR	SD	PD	NE	DCR (%)	mPFS	mFollow-Up
1	26	4	5	2	12	3	42.3%	5 (1–226)	5 (1–226)
2	20	1	4	2	8	5	35%	2.5 (0–17)	-
3	10	0	0	0	9	1	0%	-	2 (0–3)
4	6	0	1	-	4	1	16.7%	-	2.5 (0–37)
5	2	0	-	-	2	-	0%	-	4 (3–5)

CR: Complete Response; DCR: Disease Control Rate; mFollow-Up: Median Follow-Up; mPFS: Median Progression-Free Survival; N: Number of Patients; NE: Not Evaluated; PR: Partial Response; SD: Stable Disease.

**Table 5:** Systemic treatments used in the advanced setting and their outcomes.

First-Line Treatment	N = 26	mPFS
Carboplatin + paclitaxel	10	4.5 (1–108)
Carboplatin + epirubicin	4	5 (3–9)
Cisplatin + ifosfamide	3	5 (4–7)
Carboplatin + paclitaxel + dostarlimab	2	6.5 (5–8)
Ifosfamide + adriamycin/doxorubicin	2	2.5 (2–3)
Paclitaxel + bevacizumab	1	5
Gemcitabine + adriamycin	1	71
Gemcitabine + docetaxel	1	226
Cisplatin + cyclophosphamide	1	5
Dacarbazine + gemcitabine	1	2
**Second-Line treatment**	**N = 20**	**mPFS**
Dacarbazine + gemcitabine	4	3.5 (0–6)
Cisplatin + Ifosfamide	3	6 (2–8)
Carboplatin + pegylated liposomal doxorubicin	2	5.5 (1–10)
Carboplatin + paclitaxel + dostarlimab	1	17
Carboplatin + epirubicin	1	1
Carboplatin + gemcitabine + maintenance niraparib	1	8
Paclitaxel (3-weekly)	1	1
Paclitaxel (2-weekly)	1	1
Gemcitabine + docetaxel	1	1
Ifosfamide + adriamycin/doxorubicin	1	1
Pembrolizumab + lenvatinib	1	3
Oral etoposide	1	11
Epirubicin	1	2
Dostarlimab	1	0
**Third-Line Treatment**	**N = 10**	**mPFS**
Carboplatin + paclitaxel	3	1 (0–3)
Oral etoposide	2	2.5 (2–3)
Carboplatin + epirubicin	1	2
Carboplatin + gemcitabine + docetaxel	1	3
Paclitaxel Weekly + biweekly pegylated liposomal Doxorubicin	1	1
Paclitaxel + ifosfamide	1	2
Cisplatin + ifosfamide	1	0
**Fourth-Line Treatment**	**N = 6**	**mPFS**
Ifosfamide + adriamycin/doxorubicin	1	37
Carboplatin + pegylated liposomal doxorubicin	1	4
Megestrol acetate	1	3
Gemcitabine + docetaxel	1	2
Docetaxel	1	2
Trabectedin	1	0
**Fifth-Line Treatment**	**N = 2**	**mPFS**
Carboplatin + paclitaxel	1	5
Methotrexate + etoposide + bevacizumab	1	3

mPFS: Median Progression-Free Survival; N: Number of Patients.

**Table 6:** Prior publications (in chronological order) on real-world evidence of systemic treatment in GCS.

First Author, Year of Publication	Hospital, Country	N	Time Period	Population Characteristics	ChT Regimen	Results
Hoskins PJ et al., 2008 [16]	British Columbia Cancer Agency, Canada	28	From 1999 to?	Recurrent and newly diagnosed ECS	Paclitaxel 175 mg/m2 over 3 h + carboplatin (AUC 5–6) every 4 weeks for 3–6 cycles ± subsequent pelvic irradiation	Recurrent ECS: RR 55%; mPFS 12 months. Newly diagnosed ECS RR 60% mPFS 16 months.
Gonzalez Bosquet et al., 2010 [18]	Mayo Clinic Rochester, USA	121	1982–2003	Newly diagnosed ECS	MVAC, MAP, CT, VAC or CAP	5-year disease-free survival (DFS) by stage: I and II 59%; III 22%; IV 9%
Dave KS et al., 2011 [27]	Gujarat Cancer Research Institute, Ahmedabad, India	25	2000–2008	Newly diagnosed ECS	Not specified	3-year DFS 40% (for patients receiving adjuvant treatment: RT and ChT)
Anupama et al., 2013 [28]	Amrita Institute of Medical Sciences, India	20	January 2005–December 2010	ECS	Not specified except for 1 patient (cisplatin and ifosfamide)	Stage I OS 36 months. Stage II-III-IV OS 9 months.
Wallwiener et al., 2016 [19]	Germany, University of Tübingen	18	1983–2010	ECS	ifosfamide, doxorubicin, non-pegylated liposomaldoxorubicin, cisplatin, carboplatin, gemcitabine and paclitaxel—no information of the outcomes of each of the regimens	Median DFS 48.7 months; mOS 49.9months; 5-year survival rate 40 %
Guttman DM et al., 2016 [17]	4 institutions: Hospital of the University ofPennsylvania, Iowa Hospitals and Clinics, Henry Ford Hospita, Fox Chase Cancer Center, USA	118	1990–2004	Stage I and II ECS	58% adjuvant carboplatin and paclitaxel, 42% other AdjChT (not specified)	3-year OS rate 85 %; mFU 28 months, mOS 97 months
Terblanche L et al., 2022 [30]	Tygerberg Hospital, South Africa	61	1 January 2005 to 31 December 2014	ECS	Not specified	5-year PFS 17.3%; 5-year OS 19.7%
Tung JH et al., 2022 [29]	Chang Gung Memorial Hospital—Linkou Branch, China	168	June 1987 to April 2020	ECS	Only first-line regimens	mFollow-up 32 months; 5-year cancer specific survival 9.8%
Marquina G et al., 2025	Hospital Clinico san Carlos Madrid, Spain	62	1 January 1996 to 31 December 2024	Stage I–IV	Description of regimens used in all lines (first line to sixth line)	mFollow-up 20 months. mOS 24 months (20 in ECS cohort, 24 in OCS cohort) mOS ECS FIGO stage I–II 81 months; stage III 28 months; stage IV 7 months 3-year and 5-year mOS 24 months (13–34) DCR by stages

AUC: Area Under Curve; CAP: Cisplatin, Adriamycin, Bleomicyn; ChT: Chemotherapy; CT: Carboplatin, Paclitaxel; DFS: Disease Free Survival; ECS: Endometrial Carcinosarcoma; DCR: Disease Control Rate; MAP: Methotrexate, Adriamycin, Cisplatin; mFollow-Up: Median Follow-Up; mOS: Median Overall Survival. MVAC: Methotrexate, Vinblastine, Adriamycin, Cisplatin; N: Number of Patients; OCS: Ovarian Carcinosarcoma; OS: Overall Survival; PFS: Progression-Free Survival; RR: Response Rate; RT: Radiotherapy; VAC: Vincristine, Adriamycin, and Cisplatin.

## Data Availability

The original contributions presented in this study are included in the article. Further inquiries can be directed to the corresponding author.

## References

[1] Berton-Rigaud D., Devouassoux-Shisheboran M., Ledermann J.A., Leitao M.M., Powell M.A., Poveda A., Beale P., Glasspool R.M., Creutzberg C.L., Harter P. (2014). Gynecologic Cancer InterGroup (GCIG) Consensus Review for Uterine and Ovarian Carcinosarcoma. Int. J. Gynecol. Cancer.

[2] Matsuo K., Ross M.S., Machida H., Blake E.A., Roman L.D. (2018). Trends of uterine carcinosarcoma in the United States. J. Gynecol. Oncol..

[3] Rauh-Hain J.A., Diver E.J., Clemmer J.T., Bradford L.S., Clark R.M., Growdon W.B., Goodman A., Boruta D.M., Schorge J.O., del Carmen M.G. (2013). Carcinosarcoma of the ovary compared to papillary serous ovarian carcinoma: A SEER analysis. Gynecol. Oncol..

[4] Garg G., Shah J.P., Kumar S., Bryant C.S., Munkarah A., Morris R.T. (2010). Ovarian and uterine carcinosarcomas: A comparative analysis of prognostic variables and survival outcomes. Int. J. Gynecol. Cancer.

[5] Ledermann J., Matias-Guiu X., Amant F., Concin N., Davidson B., Fotopoulou C., González-Martin A., Gourley C., Leary A., Lorusso D. (2024). ESGO–ESMO–ESP consensus conference recommendations on ovarian cancer: Pathology and molecular biology and early, advanced and recurrent disease. Ann. Oncol..

[6] Oaknin A., Bosse T., Creutzberg C., Giornelli G., Harter P., Joly F., Lorusso D., Marth C., Makker V., Mirza M. (2022). Endometrial cancer: ESMO Clinical Practice Guideline for diagnosis, treatment and follow-up. Ann. Oncol..

[7] Collet L., González López A.M.G., Romeo C., Méeus P., Chopin N., Rossi L., Rowinski E., Serre A.-A., Rannou C., Buisson A. (2023). Gynecological carcinosarcomas: Overview and future perspectives. Bull. Cancer.

[8] Mirza M.R., Chase D.M., Slomovitz B.M., dePont Christensen R., Novák Z., Black D., Gilbert L., Sharma S., Valabrega G., Landrum L.M. (2023). Dostarlimab for Primary Advanced or Recurrent Endometrial Cancer. N. Engl. J. Med..

[9] Pecorelli S. (2009). Revised FIGO staging for carcinoma of the vulva, cervix, and endometrium. Int. J. Gynecol. Obstet..

[10] Berek J.S., Matias-Guiu X., Creutzberg C., Fotopoulou C., Gaffney D., Kehoe S., Lindemann K., Mutch D., Concin N. (2023). FIGO staging of endometrial cancer: 2023. Int. J. Gynecol. Obstet..

[11] Prat J. (2014). FIGO Committee on Gynecologic Oncology, Staging classification for cancer of the ovary, fallopian tube, and perito-neum. Int. J. Gynaecol. Obstet..

[12] Eisenhauer E.A., Therasse P., Bogaerts J., Schwartz L.H., Sargent D., Ford R., Dancey J., Arbuck S., Gwyther S., Mooney M. (2009). New response evaluation criteria in solid tumours: Revised RECIST guideline (version 1.1). Eur. J. Cancer.

[13] Lorusso D., Martinelli F., Mancini M., Sarno I., Ditto A., Raspagliesi F. (2014). Carboplatin-Paclitaxel versus Cisplatin-Ifosfamide in the treatment of uterine carcinosarcoma: A retrospective cohort study. Int. J. Gynecol. Cancer.

[14] Otsuki A., Watanabe Y., Nomura H., Futagami M., Yokoyama Y., Shibata K., Kamoi S., Arakawa A., Nishiyama H., Katsuta T. (2015). Paclitaxel and Carboplatin in patients with completely or optimally resected carcinosarcoma of the uterus: A phase II trial by the Japanese Uterine Sarcoma Group and the Tohoku Gynecologic Cancer Unit. Int. J. Gynecol. Cancer.

[15] Sutton G., Kauderer J., Carson L.F., Lentz S.S., Whitney C.W., Gallion H. (2005). Adjuvant ifosfamide and cisplatin in patients with completely resected stage I or II carcinosarcomas (mixed mesodermal tumors) of the uterus: A Gynecologic Oncology Group study. Gynecol. Oncol..

[16] Hoskins P.J., Le N., Ellard S., Lee U., Martin L.A., Swenerton K.D., Tinker A.V. (2008). Carboplatin plus paclitaxel for advanced or recurrent uterine malignant mixed mullerian tumors. The British Columbia Cancer Agency experience. Gynecol. Oncol..

[17] Guttmann D., Li H., Grover S., Bhatia S., Jacobson G., Elshaikh M., Sevak P., Feldman A., Lin L. (2015). The Impact of Adjuvant Therapy on Survival Endpoints in Women With Early-Stage Uterine Carcinosarcoma: A Multi-institutional Study. Int. J. Radiat. Oncol..

[18] Gonzalez Bosquet J., Terstriep S.A., Cliby W.A., Brown-Jones M., Kaur J.S., Podratz K.C., Keeney G.L. (2010). The impact of multi-modal therapy on survival for uterine carcinosarcomas. Gynecol. Oncol..

[19] Wallwiener C., Hartkopf A., Kommoss S., Joachim C., Wallwiener M., Taran F.A., Brucker S. (2016). Clinical Characteristics, Surgical Management and Adjuvant Therapy of Patients with Uterine Carcinosarcoma: A Retrospective Case Series. Geburtshilfe Und Frauenheilkd..

[20] van Rijswijk R., Vermorken J., Reed N., Favalli G., Mendiola C., Zanaboni F., Mangili G., Vergote I., Guastalla J., Huinink W.T.B. (2003). Cisplatin, doxorubicin and ifosfamide in carcinosarcoma of the female genital tract. A phase II study of the European Organization for Research and Treatment of Cancer Gynaecological Cancer Group (EORTC 55923). Eur. J. Cancer.

[21] Powell M.A., Filiaci V.L., Rose P.G., Mannel R.S., Hanjani P., Degeest K., Miller B.E., Susumu N., Ueland F.R. (2010). Phase II evaluation of paclitaxel and carboplatin in the treatment of carcinosarcoma of the uterus: A Gynecologic Oncology Group study. J. Clin. Oncol..

[22] Powell M.A., Filiaci V.L., Hensley M.L., Huang H.Q., Moore K.N., Tewari K.S., Copeland L.J., Secord A.A., Mutch D.G., Santin A. (2022). Randomized Phase III Trial of Paclitaxel and Carboplatin Versus Paclitaxel and Ifosfamide in Patients With Carcinosarcoma of the Uterus or Ovary: An NRG Oncology Trial. J. Clin. Oncol..

[23] Powell M., Bjørge L., Willmott L., Novák Z., Black D., Gilbert L., Sharma S., Valabrega G., Landrum L., Gropp-Meier M. (2024). Overall survival in patients with endometrial cancer treated with dostarlimab plus carboplatin–paclitaxel in the randomized ENGOT-EN6/GOG-3031/RUBY trial. Ann. Oncol..

[24] Ramondetta L.M., Burke T.W., Jhingran A., Schmandt R., Bevers M.W., Wolf J.K., Levenback C.F., Broaddus R. (2003). A phase II trial of cisplatin, ifosfamide, and mesna in patients with advanced or recurrent uter-ine malignant mixed müllerian tumors with evaluation of potential molecular targets. Gynecol. Oncol..

[25] Sutton G., Brunetto V.L., Kilgore L., Soper J.T., McGehee R., Olt G., Lentz S.S., Sorosky J., Hsiu J.-G. (2000). A Phase III Trial of Ifosfamide with or without Cisplatin in Carcinosarcoma of the Uterus: A Gynecologic Oncology Group Study. Gynecol. Oncol..

[26] Miller B.E., Blessing J.A., Stehman F.B., Shahin M.S., Yamada S.D., Secord A.A., Warshal D.P., Abulafia O., Richards W.E., Van Le L. (2010). A phase II evaluation of weekly gemcitabine and docetaxel for second-line treatment of recurrent carcinosarcoma of the uterus: A gynecologic oncology group study. Gynecol. Oncol..

[27] Dave K.S., Chauhan A., Bhansali R., Arora R., Purohit S. (2011). Uterine carcinosarcomas: 8-year single center experience of 25 cases. Indian J. Med Paediatr. Oncol..

[28] Anupama R., Kuriakose S., Vijaykumar D.K., Pavithran K., Jojo A., Indu R.N., Sheejamol V.S. (2013). Carcinosarcoma of the Uterus—A Single Institution Retrospective Analysis of the Management and Outcome and a Brief Review of Literature. Indian J. Surg. Oncol..

[29] Tung H.J., Chiang C.Y., Chang W.Y., Wu R.C., Huang H.J., Yang L.Y., Lin C.Y., Wang C.C., Chao A., Lai C.H. (2022). Management and Prognosis of Patients with Recurrent or Persistent/Progressive Uterine Carcinosar-coma. Curr. Oncol..

[30] Terblanche L., Botha M.H. (2022). Uterine carcinosarcoma: A 10-year single institution experience. PLoS ONE.

